# Supplementary material for: Enhanced heterogeneous ice nucleation by special surface geometry
Source: Nat Commun. 2017 May 17;8:15372. doi: 10.1038/ncomms15372 (PMC5442314; doi:10.1038/ncomms15372)
Supplement: Supplementary Information — Supplementary Figures, Supplementary Table, Supplementary Notes and Supplementary References [file ncomms15372-s1.pdf]

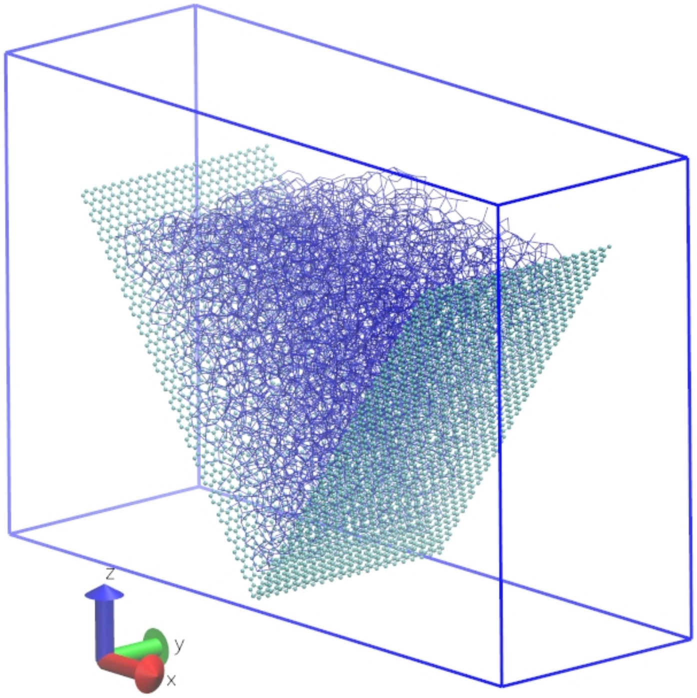

Supplementary Figure 1: **A typical simulation cell.** A periodic boundary condition is applied to the simulation cell with sufficient space left along the x and z directions. The dimension along y is about 50 Å. Carbon atoms and water molecules are colored by green and blue, respectively.

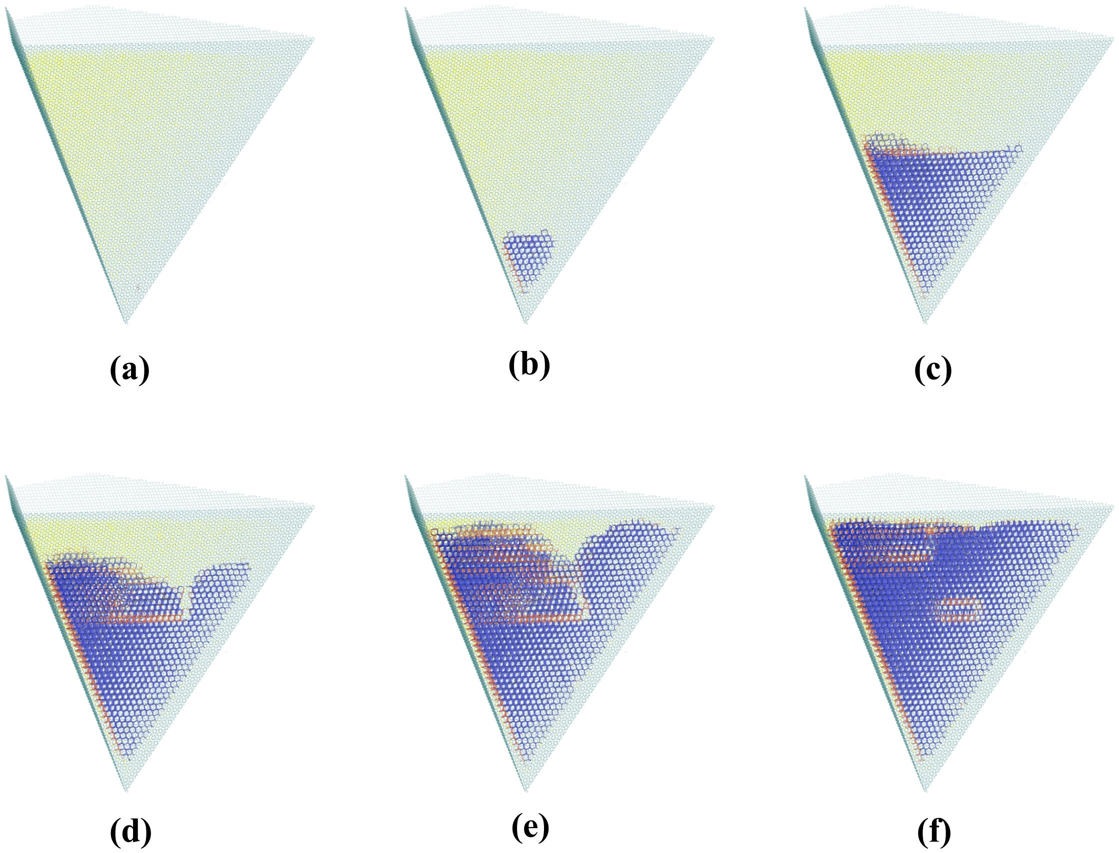

Supplementary Figure 2: **Crystallization trajectory of ice within large tetrahedral pyramid.** The spontaneous crystallization of mW ice at 240 K contains (**a**) 5, (**b**) 716, (**c**) 8,266, (**d**) 16,399, (**e**) 25,090, and (**f**) 31,705 truly ice-like water molecules, respectively. The color codes are as follows: carbon (green), liquid water (yellow), cubic ice (blue), and hexagonal ice (red).

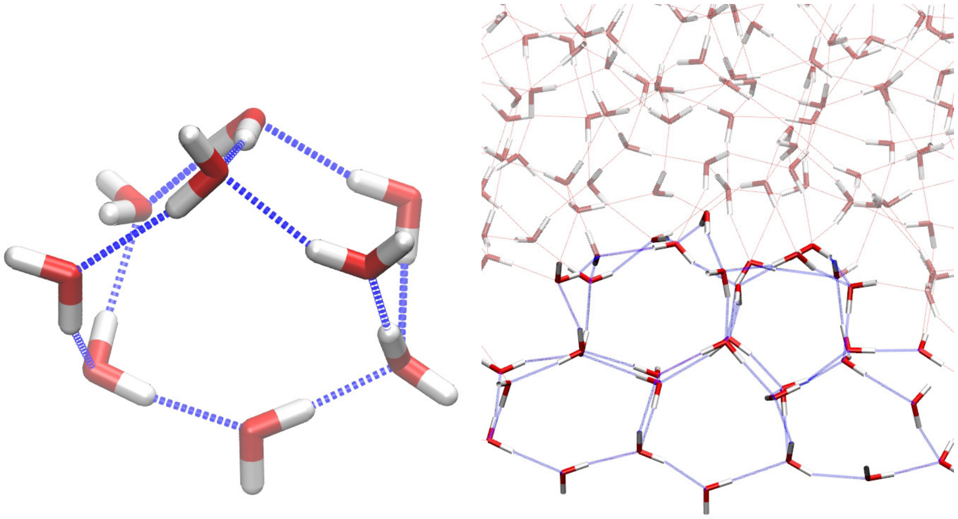

(a) (b)

Supplementary Figure 3: **Topological defect of atomistic ice at the tip of the 45° wedge.** The defect complex occurs spontaneously in the MD simulation carried out at 230 K, based on the TIP4P/Ice water model. (**a**) A wedge-shape core. (**b**) The agglomeration of topological defects. Hydrogen bonds in defects are highlighted in blue.

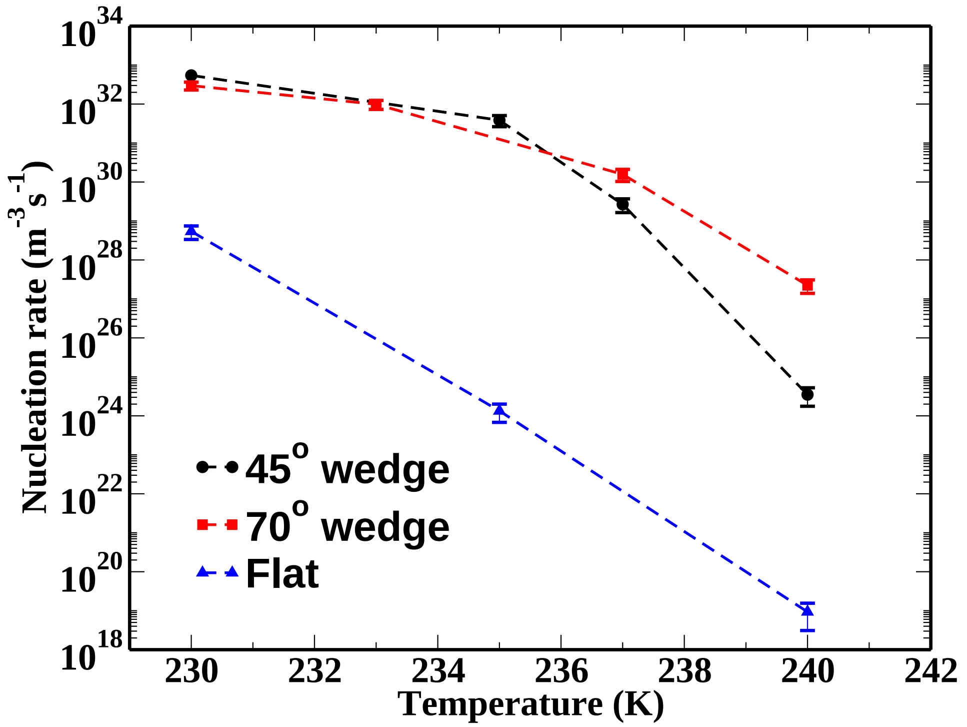

Supplementary Figure 4: **Temperature dependence of nucleation rate for mW ice.** All rates are computed by FFS, except for that at 230 K in the 45° wedge, where ice crystallization spontaneously occurs. The rates on flat graphene are from Ref. [8]. The error bars of nucleation rate are obtained in the same way as explained in Fig. 1 of the main text.

Supplementary Table 1: **Calculation of nucleation rates of mW ice crystallizing within the 43°, 45°, and 47° wedges, based on multiple direct MD simulations**

		Rate ($m^{-3}s^{-1}$)	N_C	$\sum_{i=1}^{N_C} \tau_i$ (ns)	$\sum_{j=1}^{N_{NC}} \tau_j$ (ns)
43°wedge	trial 1	4.32×10^{32}	23	234	39
	trial 2	4.27×10^{32}	15	75	106
45°wedge	trial 1	5.43×10^{32}	71	898	65
	trial 2	5.53×10^{32}	65	565	300
47°wedge	trial 1	4.06×10^{32}	44	655	143
	trial 2	3.95×10^{32}	19	200	155

Supplementary Note 1: Cubicity χ_c

The cubicity χ_c is defined as the fraction of I_c, *i.e.*, $\chi_c = N_c/N_i$, where N_c and N_i are the numbers of I_c-like and ice-like water molecules in an ice cluster. An I_c-like water molecule is numerically defined as those truly ice-like water molecules (*i.e.*, $q_6 > 0.5$) that also have a $q_3 < -0.85$. This criterion is based on the distributions of q_3 for I_h and I_c which clearly show the distinction between the two polymorphs of ice I [1].

To obtain the distribution of cubicity χ_c for fully crystallized mW ice, we fired MD shootings near the critical size for ice nucleation on a flat graphene surface at 230 K, within the 70° wedge at

230 K, and within the tetrahedral pyramid at 250 K, using the configurations collected during the FFS simulations. MD shootings yielded over 100 configurations of fully crystallized ice, which are used to compute the distribution $f(\chi_c)$, as shown in Fig. 2 in the main text.

Supplementary Note 2: Cubicity of ice crystallized in large tetrahedral pyramid

The formation of the nearly pure cubic ice I_c in tetrahedral pyramid raises an interesting question regarding the polymorph selection of ice, as both recent experiments and simulations [2] strongly suggested that the traditionally called cubic ice is in fact stacking-disordered structure, which is composed of randomly stacked hexagonal and cubic sequences. The stacking disorder of ice is attributed to the fact that the addition of cubic or hexagonal layers on existing ice basal planes is a kinetically controlled process and lacks the memory of structure underneath [3].

In contrast, the cubicity for ice crystallized in the 70° wedge and tetrahedral pyramid is found to be significantly higher, with the pyramid case being even close to 100%. Since the tetrahedral pyramid contains around 6,000 water molecules, it is of interest to understand whether the picture holds for a larger system. To do this, we created a large tetrahedral pyramid, containing 38,648 water molecules and 29,427 carbon atoms. As in the small system, the large tetrahedral pyramid is also found to induce spontaneous ice crystallization at 240 K, as shown in Supplementary Figure 2. The fully crystallized configuration yields a cubicity of 86%, with only a few stacking faults near the top of the pyramid. The significantly higher cubicity of ice crystallized within tetrahedral pyramid can be attributed to the structural template created by the special wedge geometry that

favors diamond cubic structure. In other words, the growth of ice basal planes in pyramid does feel the existence of three wedge contact lines. Certainly, when the size of wedge becomes large enough, such influence would gradually vanish and ice growth would be controlled by kinetics again. Further studies are needed to understand how large ice could maintain high cubicity before returning to regular stacking disordered structure and whether this volume of cubic ice can be experimentally detectable, *e.g.*, through X-ray diffraction.

Supplementary Note 3: Topological defects with atomistic water models

Ice crystallization within the 45° wedge is found to be initiated by the formation of a unique wedge-shape core (WC). Although our study is carried out using the coarse-grained mono-atomic water mW model, the same defect is also previously identified in the topological analysis of supercooled water [4] based on the six-site water model [5] that contains explicit hydrogen bond. Thus it is highly likely that the appearance of the WC structure in nucleation is a generic attribute of tetrahedral network. To confirm this, we created a 45° wedge and filled it with 1,286 TIP4P/Ice water molecules [6]. The carbon-oxygen interaction is represented by the Lennard-Jones potential:

$$U(r_{ij}) = 4\epsilon\left[\left(\frac{\sigma}{r_{ij}}\right)^{12} - \left(\frac{\sigma}{r_{ij}}\right)^{6}\right],$$

with $\epsilon = 0.13$ Kcal/mol, $\sigma = 3.2$ Å. The choice of these parameters is based on the corresponding mW model for water-carbon interaction, to ensure that two sets of force fields describe the same energy and length scales.

The system is equilibrated for 5 ns and then switched to NVT ensemble for 40 ns at 230 K. Although no spontaneous ice crystallization is observed during this period, this is not unexpected because nucleation rate is extremely sensitive to the difference in force fields. For example, although both mW and TIP4P/Ice water models have very similar equilibrium ice melting temperatures, the calculated homogeneous ice nucleation rates for the two models [1,7] differ by seven orders of magnitude at 230 K. Despite the absence of ice crystallization, the simulation indeed sees the frequent formation of the same type of topological defect structures near the wedge contact line, as shown in Supplementary Figure 3.

Supplementary Note 4: Temperature dependence of ice nucleation rate in 45° and 70° wedge

The enhancements of ice nucleation within the 45° and the 70° wedges are found to be attributed to different nucleation mechanisms, *i.e.,* structural match to topological defects for the 45° wedge, and structural match to perfect ice lattice for the 70° wedge, respectively. It is of interest to understand how their ice nucleation efficiencies vary with temperature. We use the calculated nucleation rate as the key metric to address this question. As shown in Supplementary Figure 4, the calculated rates for two special geometries show a crossover near 236 K, below which the 45° wedge yields a slightly higher ice nucleation rate than that for the 70° wedge. At higher temperature, the 70° wedge exhibits a stronger ice nucleation efficiency than the 45° wedge and this preference appears to further increase with temperature. Although a more detailed, quantitative future study is needed to elucidate this temperature dependence, the trend is qualitatively consistent with the previous study [4] that suggests the topological fragments become increasingly stable and abundant as

temperature decreases. In light of Ostwald step rule, this makes them kinetically favorable for crystallization pathways at low temperature. On the contrary, the stability and the propensity of these defects diminish at higher temperature, making this alternative nucleation route less favorable than a direct pathway in 70° wedge. Nevertheless, this pathway is still found to be much more favorable than that on a flat surface at high temperature [8] (Supplementary Figure 4), because ice nucleation within the 45° wedge is still facilitated through a match (albeit a match to a non-crystalline unit) by two dimensions rather than by only one dimension on a planar surface.

Supplementary References

1. Li, T., Donadio, D., Russo, G. & Galli, G. Homogeneous ice nucleation from supercooled water. *Phys Chem Chem Phys* **13**, 19807–19813 (2011).

2. Malkin, T. L. *et al.* Stacking disorder in ice I. *Phys Chem Chem Phys* **17**, 60–76 (2014).

3. Hudait, A., Qiu, S., Lupi, L. & Molinero, V. Free energy contributions and structural characterization of stacking disordered ices. *Phys Chem Chem Phys* **18**, 9544–9553 (2016).

4. Matsumoto, M., Baba, A. & Ohmine, I. Topological building blocks of hydrogen bond network in water. *J Chem Phys* **127**, 134504 (2007).

5. Nada, H. & van der Eerden, J. P. J. M. An intermolecular potential model for the simulation of ice and water near the melting point: A six-site model of H2O. *J Chem Phys* **118**, 7401–7413 (2003).

6. Abascal, J., Sanz, E., Fernandez, R. & Vega, C. A potential model for the study of ices and amorphous water: TIP4P/Ice. *J Chem Phys* **122**, 234511 (2005).

7. Haji-Akbari, A. & Debenedetti, P. G. Direct calculation of ice homogeneous nucleation rate for a molecular model of water. *Proc Natl Acad Sci USA* **112**, 10582–10588 (2015).

8. Cabriolu, R. & Li, T. Ice nucleation on carbon surface supports the classical theory for heterogeneous nucleation. *Phys Rev E* **91**, 052402 (2015).
